# Knockdown of hnRNPA1 Promotes NSCLC Metastasis and EMT by Regulating Alternative Splicing of LAS1L exon 9

**DOI:** 10.3389/fonc.2022.837248

**Published:** 2022-06-23

**Authors:** Peng Han, Peng Cao, Jiaqi Yue, Kangle Kong, Shan Hu, Yu Deng, Lequn Li, Fan Li, Bo Zhao

**Affiliations:** ^1^ Department of Thoracic Surgery, Tongji Hospital, Tongji Medical College, Huazhong University of Science and Technology, Wuhan, China; ^2^ Thoracic Surgery Laboratory, Department of Thoracic Surgery, Tongji Hospital, Tongji Medical College, Huazhong University of Science and Technology, Wuhan, China

**Keywords:** lung cancer, hnRNPA1, alternative splicing, Las1l, metastasis

## Abstract

Tumor metastasis is still an insurmountable obstacle in tumor treatment. Lung cancer represents one of the most common malignancies with high morbidity worldwide. hnRNPA1 has been reported to be involved in the regulation of tumor metastasis, while its specific role in tumor metastasis seems to be controversial and its molecular mechanism in lung cancer metastasis remains to be further elucidated. In this study, we confirmed that knockdown of the hnRNPA1 led to enhanced migration, invasion and EMT transition in lung cancer cells. Bioinformatics analysis of the GSE34992 dataset revealed that hnRNPA1 may regulate the alternative splicing (AS) of LAS1L exon 9. Further AGE assays and RIP assays revealed that hnRNPA1 can directly bind to the LAS1L pre-mRNA to inhibit the splicing of LAS1L exon 9. The RNA pull-down assays showed that hnRNPA1 can specifically bind to the two sites (UAGGGU(WT1) and UGGGGU(WT3)) of LAS1L Intron 9. Further Transwell assays indicated that the expression ratio of LAS1L-L/LAS1L-S regulated by hnRNPA1 can further promote the migration, invasion and EMT transition in lung cancer cells. Moreover, hnRNPA1 expression showed significant heterogeneity in lung cancer tissues, which may contain new research directions and potential therapeutic targets. Our results indicate that hnRNPA1 can affect the metastasis of lung cancer cells by modulating the AS of LAS1L exon 9, highlighting the potential significance of hnRNPA1 in lung cancer metastasis.

**Graphical Abstract:**
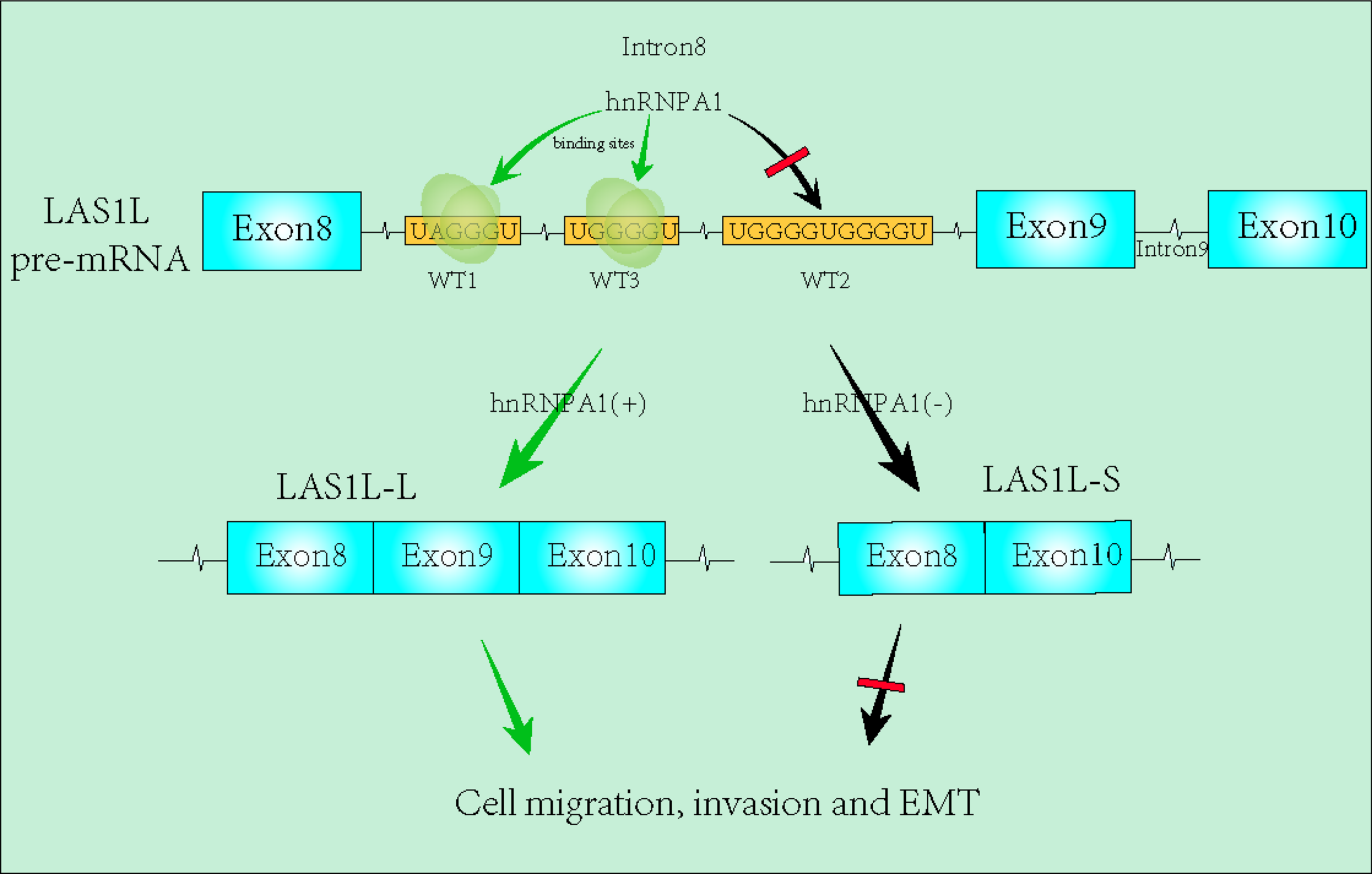


## Introduction

Tumor metastasis remain one of the leading causes of lung cancer mortality, with more than 90% of lung cancer patients dying from tumor metastasis (1). Unfortunately, a large number of lung cancer patients are still diagnosed in the middle and later stages, which means a higher incidence rate of metastasis (1, 2). The abnormal activation of epithelial–mesenchymal transition (EMT) has reported to be one of the crucial mechanisms that facilitates cancer metastasis (3). Despite numerous studies, our understanding of lung cancer metastasis is still insufficient. Further molecular mechanism studies are urgently needed.

Alternative splicing refers to any pre-mRNA splicing process by which part of exon/intron from a nascent transcript is differentially included in the mature mRNA. This specific splicing regulation, which occurs throughout the process of cell development and functional phenotype, extremely contributes to the diversity of mRNA and proteins (4, 5). Interestingly, AS exerts an even more unexpected effect in tumor. As shown in a comprehensive analysis from 8,705 patients, tumors have up to 30% more AS events than normal samples (6). The proteins involved in AS regulation can generally be divided into three parts: hnRNPs family, SR family and other tissue-specific RNA binding proteins (7). Generally speaking, SR proteins promote the splicing events at adjacent splicing sites by interacting with exonic splicing enhancers, whereas hnRNPs proteins also inhibit the splicing events by interacting with exonic splicing silencer, and tissue-specific RNA splicing factors can act as activators or suppressors (8). hnRNPA1 is one of the most abundant RNA binding proteins in the hnRNPs family, and its crucial function in tumor is particularly noticeable. It’s reported that hnRNPA1 can promote tumor aerobic glycolysis by regulating mutually exclusive splicing of pyruvate kinase (PKM) exons 9 and 10 (9), which partially revealed the molecular mechanism of Warburg effect and suggested the importance of hnRNPA1 in tumor genesis and development. Except for metabolic regulation, hnRNPA1 is also involved in the metastasis of gastric cancer (10), liver cancer (11), bladder cancer (12) and pancreatic cancer (13). Interestingly, some studies have shown that hnRNPA1 can promote tumor metastasis and EMT, while others drawn completely opposite conclusions (14–16). Further studies should be carried out to elucidate its molecular mechanisms.

In our analysis, we found that hnRNPA1 can further regulate the AS of LAS1L pre-mRNA. LAS1L, as one of the major members of 5FMC (Five Friends of Methylated Chtop), has been reported to be involved in ribosome biogenesis, and is also associated with TP53 cell cycle and autophagy (17–19). The crucial role of LAS1L in tumor progression is being revealed. A recent study from Dr. Samant also showed that knockdown of LAS1L leads to a reduction in proliferation and metastatic potential of triple negative breast cancer cells (20). However, studies on the AS regulation of LAS1L in tumor have not been reported. Our analysis of the NCBI database identified that there are 14 exons in human LAS1L gene, among which exon 9 is differentially expressed in tumor tissues (https://bioinformatics.mdanderson.org/TCGASpliceSeq/singlegene.jsp). Whether deletion of exon 9 will the lead to functional or structural differences of these two LAS1L isoforms has aroused great attention from us.

In this study, we demonstrated the effects of hnRNPA1 on NSCLC metastasis and its molecular mechanism, and found that knockdown of hnRNPA1 can significantly promote lung cancer metastasis and EMT transition by inhibiting the exon skipping events of LAS1L exon 9. Notably, hnRNPA1 directly binds to the LAS1L intron 8 to promote the skipping of LAS1L exon 9, while the regulated LAS1L-L can further promote the metastasis and EMT transition of NSCLC. Overall, we reveal a novel mechanism for lung cancer metastasis.

## Materials and Methods

### Cell Culture

293T, A549 and H1299 were provided by Cobioer Biosciences (Nanjing, China). Lung cancer cell lines were cultured in RPMI-1640 (Hyclone, Omaha, NE, USA). All media was supplemented with 10% fetal bovine serum (FBS; Gibco, Grand Island, NY, USA) and 100 U/mL penicillin/streptomycin (Hyclone, Logan, UT, USA). All cells were cultured at 37°C in an atmosphere containing 5% CO2. All cell lines were passaged for less than 8 weeks after thawing.

### Plasmid Construction

The short hairpin RNA (shRNA) plasmid and the over-expression plasmid were gifted from the Laboratory of Biliopancreatic Surgery, Tongji Hospital, Tongji Hospital, Huazhong University of Science and Technology. For construction of shRNA vectors, the shRNA primers of Las1l -S, Las1l -L, and Las1l were designed (Table S1). The forward and reverse primers were gradient annealed at 95°C to 25°C (-0.5°C/30 sec). Subsequently, double-strand oligonucleotides were inserted into the sites Age I (Thermo Fisher Scientific, USA) and EcoR I (Thermo Fisher Scientific, USA) of the double digestion pCDH-EF1-copGFP plasmid. For construction of over-expression vectors, pHAGE-puro plasmid was digested with MIuI (Thermo Fisher Scientific, USA) and BmtI (Thermo Fisher Scientific, USA), and the linearized plasmid was homologously ligated with the Las1l-L-Flag (ORF region) to construct a pHAGE-Las1l-L-Flag plasmid. The recombinant plasmid was transformed into DH5α (TsingKe, China) competent cells and overnight cultured on ampicillin-containing of plates. Correct plasmid construction was determined by nucleic acid sequencing. For construction pcDNA3.1-LAS1L-minigene vectors, the Las1l-minigene genomic sequences spanning exons 8 to 10 were synthesized and was cloned into the pcDNA3.1 (+) vector (TsingKe, China). All Plasmid were extracted by usingMolPure^®^ Endo-free Plasmid Mini Kit (Yeasen, China). All the primers used in this study were synthesized by WuHan Tsingke Biological Company.

### Construction of Mutant Plasmid

To construct of the pcDNA3.1-Las1l-minigene mutant plasmid, mutant primers Las1l-M1-F and Las1l-M1-R (Las1l-M2-F/R or Las1l-M3-F/R) were designed. The pcDNA3.1-Las1l-minigene plasmid was used as template for PCR amplification reaction. Subsequently, 50 μL of PCR product was mixed with 1 μL Dpn I (Vazyme, USA) and then reacted at 37°C for 90min. Then, homologous recombination was performed on the mutant site of the digested product. All those reagents were from Mut Express II Fast Mutagenesis Kit V2 originated from Vazyme Biological Company. Then, 5 μL of the recombinant plasmid was transformed into DH5α competent cells and cultured on ampicillin-containing of plates overnight. Correct plasmid construction was extracted after verification by nucleic acid sequencing.

### Transfection and Retrovirus Infection

Together with recombinant plasmid, psPAX, and pMD2G plasmids, recombinant lentiviral vectors were transfected into 293T cells for 2 days. After the lentivirus is filtered, target cells were infected with supernatant for 6h. Then, the transfected cells were treated with puromycin for 2 weeks. After the efficiency of overexpression or depletion plasmids was confirmed, surviving cells were used for further experiments. The qPCR and western blot assay were used to confirm the efficiency of overexpression or depletion plasmids, stable transfected cells were used for further experiments.

### Western Blot Analysis

The RIPA lysis buffer (50 mM Tris pH 7.4, 150 mM NaCl, 1% Triton X-100, 1% sodium deoxycholate, and 0.1% SDS) was used to lysis cells. Cell lysates were separated by 10% SDS-PAGE and transferred to the PVDF membrane (Merck Millipore, Darmstadt, Germany). Then, the PVDF membrane was blocked by TBST buffer (TBS containing 0.1% Tween 20 and 5% non-fat dry milk) for 60 min. Next, will be incubated with primary antibody overnight. HnRNPA1 primary antibody (D21H11), E-Cadherin primary antibody (24E10), N-Cadherin primary antibody (D4R1H), Vimentin primary antibody (D21H3) were purchased from Cell Signaling Technology Company. β-actin primary antibody (2D4H5) was purchased from Proteintech Company. The blots were then performed using an enhanced chemiluminescent detection system (Tanon 5200, Shanghai, China). Image J software (National Institutes of Health, USA) was used to quantify the grayscale of bands.

### Semi-Quantitative Reverse Transcriptase PCR (RT-PCR) and Quantitative Real-Time PCR (qPCR)

TRIzol reagent (Yeasen, China) was used to extract the total RNAs of cells or tissues, and the mRNA was reverse transcribed into cDNA using an cDNA Synthesis Kit (Yeasen, China). Semi-quantitative RT-PCR was performed with 2×Hieff^®^ HotStart PCR Genotyping Master Mix (Yeasen, China). The inclusion/exclusion forms of exon9 of LAS1L were detected by RT-PCR, and these two isoforms were depicted on 2% agarose gels. β-actin was used as internal control. RT-qPCR was performed using Hieff UNICON^®^ Power qPCR SYBR Green Master Mix (Yeasen, China) *via* Applied Biosystems (Vilnius, Lithuania). All primers used in this study were listed in **Supplementary table 1 (Table S1** ***).***

### Wound-Healing Assays

For wound-healing assays, the cells were seeded in 6-well plates. When reached 100% fusion, the cells were scratched onto the plate using 200 μL pipette tip, and then cultured in serum-free 1640 medium. For each sample, 5 areas were randomly selected and observed under 10 x 10 magnification at 0 and 24 h, respectively. The relative distance of wound closure was used to evaluate cell migration ability.

### Transwell Assays

For migration assays, about 8 × 10^4 of cells were seeded in the upper chambers of 24-well Transwell plates (Corning, USA) in FBS-free medium, and RPMI-1640 medium with 10% FBS was deposited in the lower chambers. For invasion assay, Transwell membranes were coated with Matrigel (Corning, USA) prior to seeding cells. After about 24-36 h, cells were fixed in 4% paraformaldehyde for 20 min and stained for 20 min in crystal violet. Swab the cells in the upper side of the chambers completely. The relative number of passed cells represented the abilities of migration or invasion. Multiple random fields were visualized under a wide-field fluorescent microscope (Carl Zeiss, Baden-Württemberg, Germany) at 200× magnification.

### RNA Immunoprecipitation (RIP)

According to the manufacturer’s instructions, RIP experiments were performed using the Magna RIP Kit (17-701, Millipore) to clarified the binding ability of hnRNPA1 to Las1l pre-RNA.

### RNA Pull-Down Assay

RNA pull-down experiments were performed using Pierce™ Magnetic RNA-Protein Pull-Down Kit (Thermo Scientific, USA). The biotinylated Las1l RNA (Tsingke, China) probe was synthesized by WuHan Tsingke Biological Company. Then, 50 μl streptavidin magnetic beads were used to capture the biotin-labeled RNA. Incubate the streptavidin magnetic bead mixture with the protein lysate at 4° C for 45 min. The RNA-Binding Protein Complexes were eluted and analyzed by Western blot.

### Statistical Analysis

Statistical analyses were performed using Prism V6 software (GraphPad, La Jolla, CA, USA). The data were presented as the mean ± SEM. A two-sided Student’s t-test was performed to compare the difference between the two groups. The data in the bar graphs are presented as the mean ± SEM. Differences were considered statistically significant at p <.05. P < 0.05, P < 0.01 and P < 0.001 were marked as ‘*’, ‘**’, ‘***’.

## Results

### Knockdown of hnRNPA1 Promotes NSCLC Metastasis and EMT

**Figure 1:**
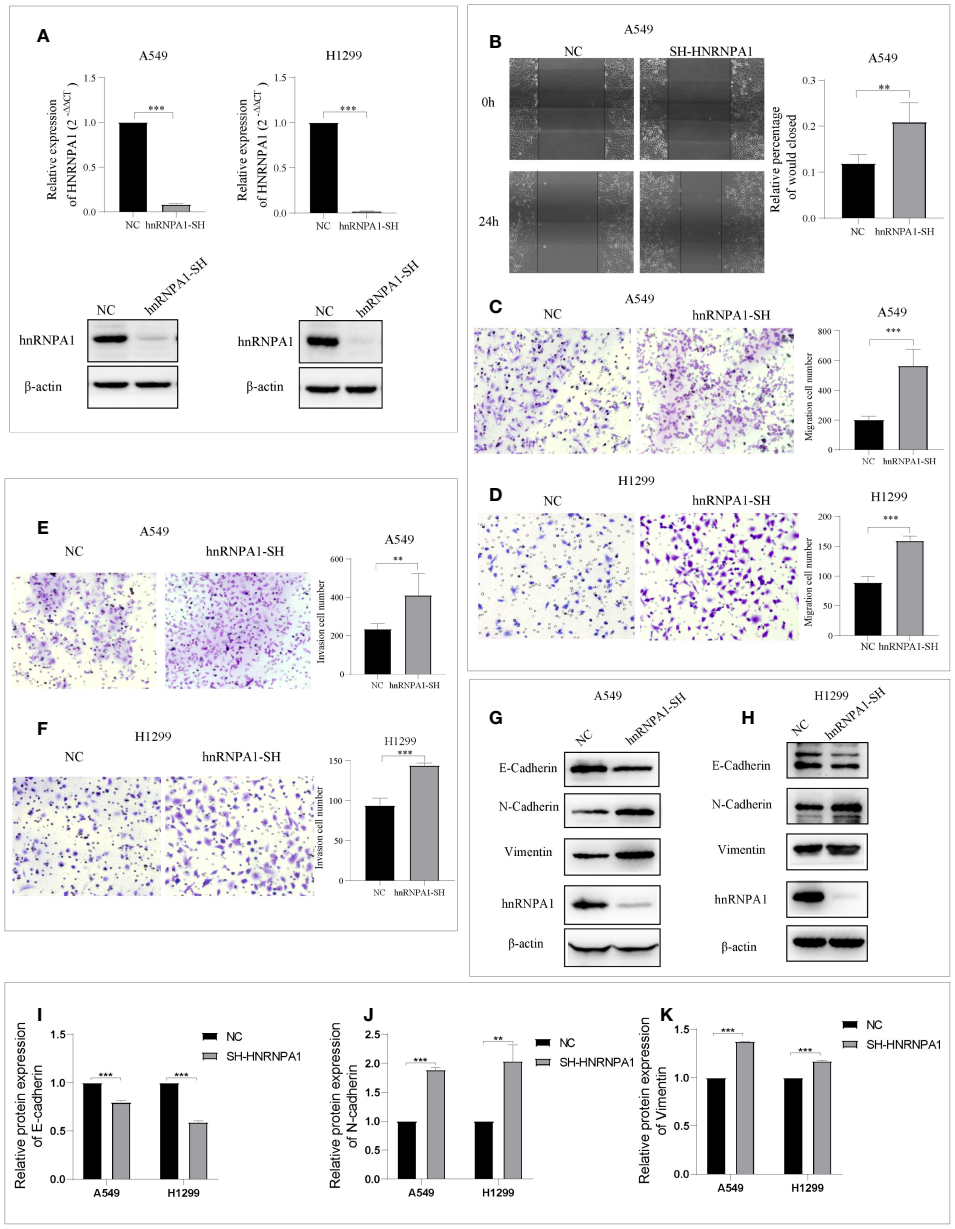
Knockdown of hnRNPA1 promotes NSCLC metastasis and EMT. **(A)** Verification of HNRNPA1 knockout efficiency. **(B–D)** Wound-healing assays and Transwell cell migration assays showed that knockdown of hnRNPA1 significantly promoted the migration of A549 and H1299 cells. **(E–F)** Transwell cell invasion assays showed an enhanced invasion ability after knockdown of hnRNPA1 in A549 and H1299 cells. **(G–K)** HNRNPA1 depletion accelerated the transition of EMT. Data are means ± SD (**P < 0.01, ***P < 0.001).

HnRNPA1 has attracted significant attention as a promising tumor-related regulatory factors of alternative splicing. However, its crucial role in NSCLC metastasis remains controversial and to be further studied. To further explore whether hnRNPA1 affects lung cancer metastasis and its potential molecular mechanism, we firstly designed an independent shRNA targeted against hnRNPA1 (**Table S2**), and verified the efficiency in the NSCLC cell lines of A549 and H1299 (**Figure 1A**). Then, we assessed the effects of hnRNPA1 depletion on cell migration. Wound-healing assays and Transwell cell migration assays all showed that knockdown of hnRNPA1 significantly promoted the migration of A549 and H1299 cells (**Figures 1B–D**). Similarly, Transwell cell invasion assays showed that knockdown of hnRNPA1 significantly promoted the invasion of A549 and H1299 cells (**Figures 1E–F**). Previous studies have shown that hnRNPA1 may regulate tumor metastasis by regulating EMT-related molecules, so we sought to determine whether hnRNPA1 regulates NSCLC metastasis through regulating the EMT process. Western blot assays (**Figures 1G–H**) showed that hnRNPA1 depletion inhibited the expression of E-cadherin (**Figure 1I**) and promoted the expression of N-cadherin (**Figure 1J**) and Vimentin (**Figure 1K**), which suggested that hnRNPA1 might promote tumor metastasis by accelerating the transition of mesenchymal phenotypic in NSCLC.

### LAS1L exon 9 Is Subject to the Regulation by hnRNPA1

**Figure 2:**
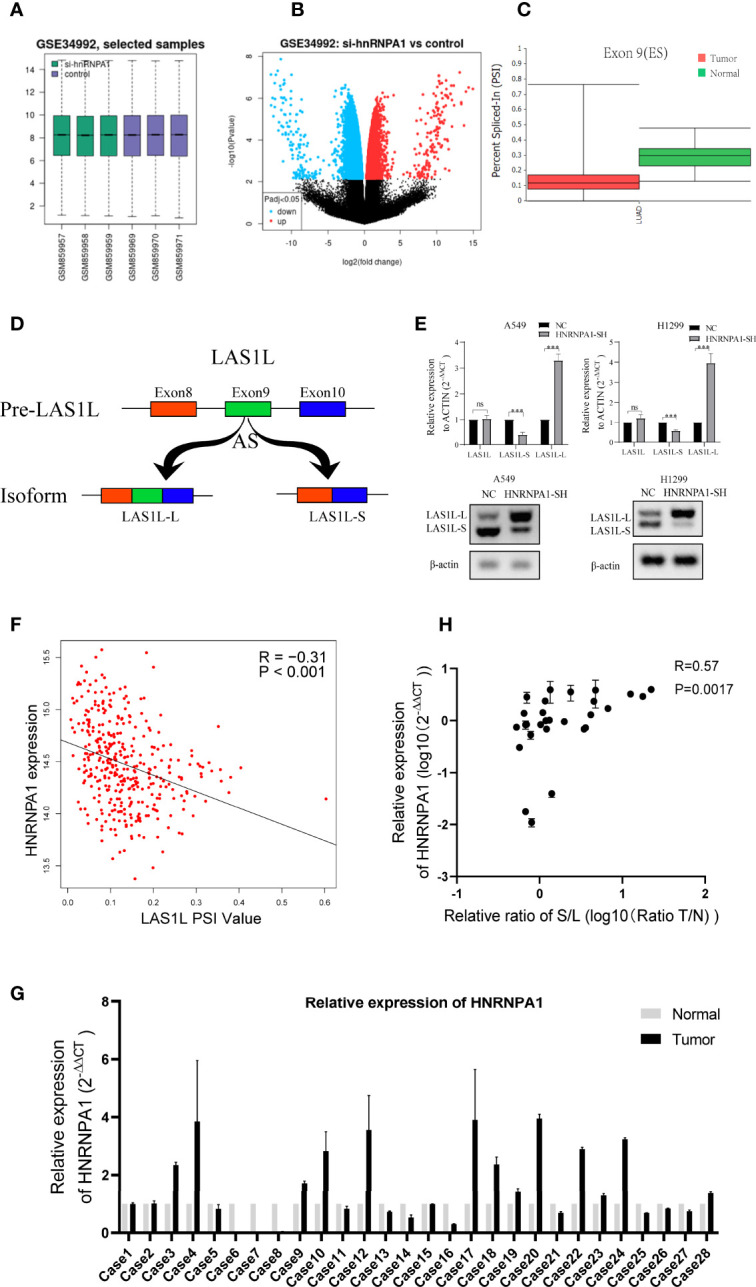
LAS1L exon 9 is subject to the regulation by HNRNPA1. **(A)** Boxplot of si-hnRNPA1 group and control group in GSE34992 dataset. **(B)** Volcanic maps for differential expression analysis of splicing events in si-hnRNPA1 group and control group. **(C)** Relative expression of LAS1L exon 9 on *TCGA SpliceSeq* in lung cancer tissues. **(D)** Illustration of LAS1L pre-mRNA, LAS1L long and short isoforms. **(E)** qPCR assays and agarose gel electrophoresis (AGE) showed that knockdown of hnRNPA1 significantly inhibited the LAS1L exon 9 skipping event in A549 and H1299 cell lines. **(F)** Pearson correlation analysis of the expression level of hnRNPA1 and the PSI value of LAS1L exon9 from TCGA datasets showed that hnRNPA1 can promote the exon skipping of LAS1L exon9 (r = -0.31, P<0.001). **(G)** qPCR assays indicated that HNRNPA1 was differentially expressed in 29 paired of NSCLC tissue specimens. **(H)** The expression ratio of LAS1L-S/L has a moderate correlation with the expression of hnRNPA1 in tissue specimens. Data are means ± SD (**P < 0.01; ns, P >0.05).

It has been clearly reported that hnRNPA1 has crucial roles in the regulation of many cellular AS events. In order to identify whether there are any other transcripts involved in the regulation of hnRNPA1 in NSCLC metastasis, we searched and analyzed the GSE34992 dataset (21), which focused on downstream AS events associated with hnRNPs in 293T cells. Spliced differential expression analysis was performed between hnRNPA1 knockdown groups and control groups in the GSE34992 dataset (**Figures 2A–B**). Then, the top 100 spliced differentially expressed genes were selected (**Table S3**). To identify tumor-specific differential splicing genes, we compared the splicing events of these genes in the *TCGA SpliceSeq* database and only to found that the exon 9 skipping of LAS1L was specific in lung cancer compared to normal tissue (**Figure 2C**). Depending on exclusion/inclusion of exon 9, LAS1L can generate two transcript variants (LAS1L-S and LAS1L-L) regulated by splicing factors (**Figure 2D**). Further qPCR and agarose gel electrophoresis (AGE) assays showed that knockdown of hnRNPA1 significantly inhibited the LAS1L exon 9 skipping event in A549 and H1299 cell lines (**Figure 2E**). Consistent results were also confirmed in the TCGA dataset. Then, we downloaded the percent-splice-in (PSI) data of LAS1L exon9 from *TCGA SpliceSeq (* 22 *)*. PSI is the ratio of normalized read counts indicating inclusion of a transcript element over the total normalized reads for that event (both inclusion and exclusion reads). Pearson correlation analysis of the expression level of hnRNPA1 and the PSI value of LAS1L exon9 also indirectly indicated that hnRNPA1 might promote the exon skipping of LAS1L exon9 (*r* = -0.31, *P*<0.001) (**Figure 2F**).

Similarly, Pearson’s correlation analysis was also performed between common 64 splicing proteins and the LAS1L PSI values (**Table S4**). Then, the top 10 splicing proteins were selected for further verification (**Figure S1A**). The independent shRNA targeted against each splicing protein were designed (**Table S2**) and verified (**Figure S1B**). To further verify the splicing ability of these proteins on LAS1L exon 9, qPCR assays were performed and the results showed that those proteins had no significant (fold change≥2 or ≤0.5) splicing regulatory ability on LAS1L exon 9 except hnRNPA1 protein (**Figure S1C**). To further clarify the expression of LAS1L exon 9 in NSCLC clinical samples, a total of 58 samples including 29 NSCLC tumor samples and 29 corresponding normal samples were randomly selected from the specimen bank. Semi-quantitative RT-PCR analysis and AGE assays were conducted to detect the expression ratio of LAS1L-S and LAS1L-L (**Figure S2**). RT-qPCR was used to detect the mRNA expression level of hnRNPA1 (**Figure 2G**), and the pearson’s correlation between hnRNPA1 expression and LAS1L-S/L ratio was then calculated. The results also showed that hnRNPA1 may promote the exon skipping of LAS1L exon9(*r* = 0.57, *P*=0.0017) (**Figure 2H** ***)***, which is consistent with the above results.

### LAS1L-L But Not LAS1L-S Promoted Metastasis and Mesenchymal Phenotype

**Figure 3:**
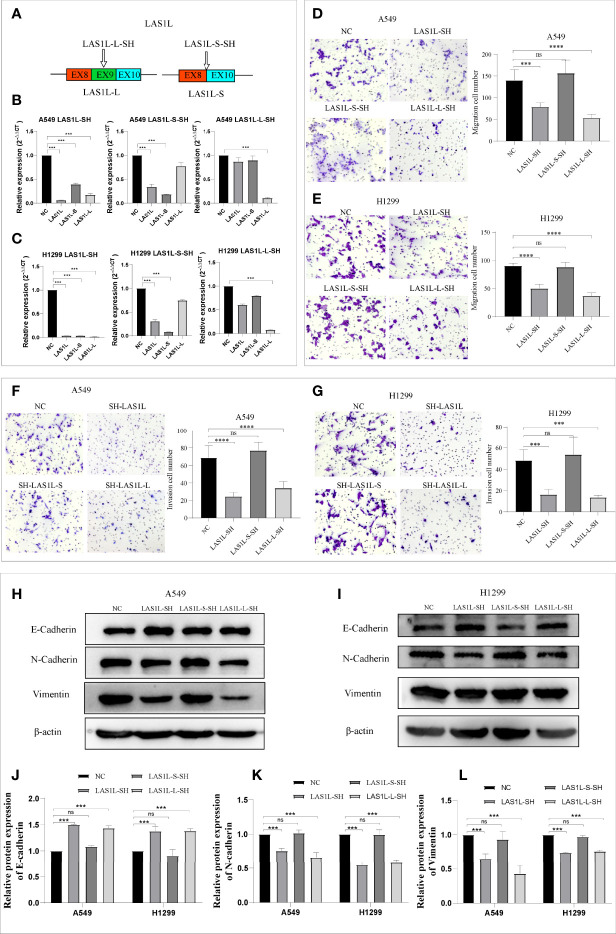
LAS1L-L but not LAS1L-S promoted metastasis and mesenchymal phenotype. **(A)** Illustration of LAS1L long and short isoforms; the arrow indicates the target site of the SH-RNA, and half of the LAS1L-S-SH target site is located in exon 8 and half in exon 10. **(B–C)** qPCR assays were used to verify the specific knockdown efficiency of LAS1L, LAS1L-S and LAS1L-L isoforms in A549 and H1299 cell lines. **(D–G)** Transwell assays showed that knockdown of LAS1L and LAS1L-L inhibited the migration and invasion ability of A549 and H1299 cells, while LAS1L-S depletion had no significant effects. **(H–I)** Western blot assays showed that LAS1L and LAS1L-L depletion inhibited the expression of N-cadherin**(K)** and Vimentin**(L)** and promoted the expression of E-cadherin **(J)** to further accelerate the EMT transition. Data are means ± SD (***P < 0.001, ****P < 0.0001; ns P > 0.05).

The above observations suggest that hnRNPA1 depletion promotes NSCLC metastasis and EMT, and promotes the expression of LAS1L-L, so we sought to determine whether the splicing variation of LAS1L exon 9 also affects metastasis and EMT of NSCLC. We firstly designed an independent shRNA to specifically knockdown the LAS1L-S isoform, and a shRNA targeted against exon 9 for isoform-specific knockdown of LAS1L-L (**Figure 3A** ***)***, and then verified their efficiency (**Figures 3B–C**). We next assessed the effects of LAS1L isoform depletion on cell migration and invasion. Transwell cell migration/invasion assays showed that knockdown of LAS1L and specific knockdown of LAS1L-L significantly inhibited the migration and invasion of A549 and H1299 cells, while exist no significant effects when knocking down the LAS1L-S isoform (**Figures 3D–G**). Similarly, we posited that the two LAS1L isoforms caused by exon9 skipping might have different effects on the transition of mesenchymal phenotypic in NSCLC. Further western blots assays showed that LAS1L or LAS1L-L knockdown cells showed a significant reduction of mesenchymal markers N-cadherin (**Figure 3K**) and Vimentin (**Figure 3L**) and a more pronounced increase in the epithelial marker E-cadherin (**Figures 3H–J**).

**Figure 4:**
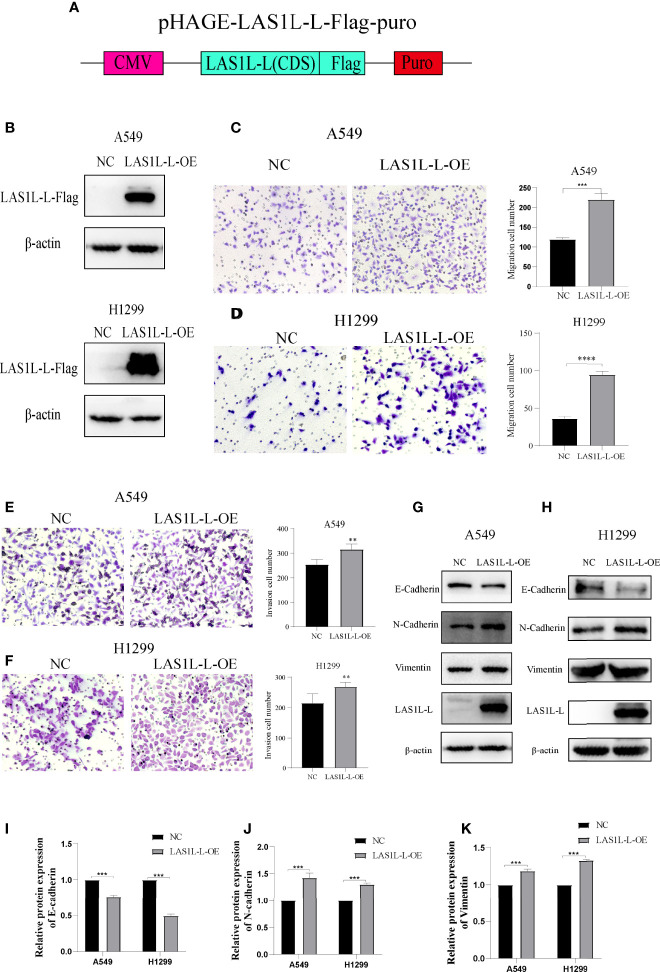
Over-expression of LAS1L-L can promote metastasis and mesenchymal phenotype. **(A)** Graphical representation of the LAS1L-L-Flag overexpression system. **(B)** Verification of LAS1L-L-Flag over-expression efficiency in A549 and H1299 cells. Transwell assays showed that over-expression of LAS1L-L can promote the migration **(C– D)** and invasion **(E–F)** ability of A549 and H1299 cells. **(G–K)** Western blot assays showed that LAS1L-L over-expression promote the EMT transition. Data are means ± SD (** P < 0.01, ***P < 0.001, ****P < 0.0001.).

The above results indicate that the LAS1L may regulate tumor metastasis ability through LAS1L-L, while LAS1L-S has no significant effect. To further verify the metastatic potential of LAS1L-L, we constructed LAS1L-L overexpression plasmid attached with FLAG fusion protein (**Figure 4A**) and then verified their protein expression efficiency in A549 and H1299 cell lines (**Figure 4B**). Next, we assessed the effects of LAS1L-L isoform on cell migration and invasion through transwell cell migration/invasion assays. The results showed that overexpression of LAS1L-L can promote the migration and invasion of A549 and H1299 cells (**Figures 4C–F**). In addition, overexpression of LAS1L-L can also promote the transition of EMT in NSCLC (**Figures 4G–K**). These data indicate that the proportion of LAS1L-L isoform is correlated with tumor metastasis ability.

### hnRNPA1 Enhances AS of LAS1L exon 9 Through Its Interaction With Upstream Intron 8 Sequences

**Figure 5:**
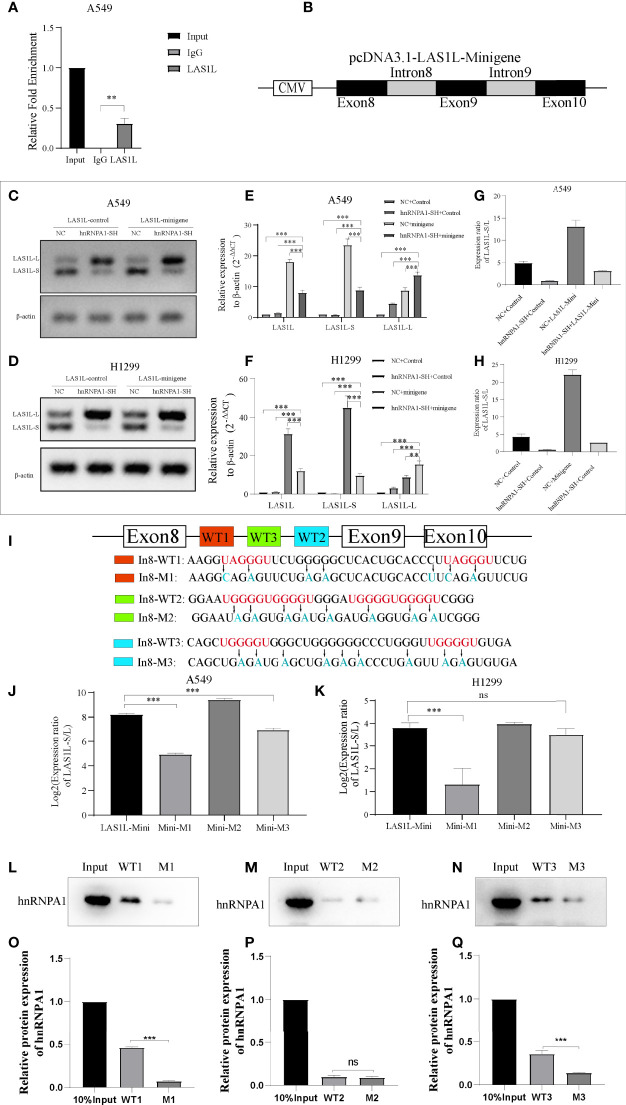
hnRNPA1 enhances AS of LAS1L exon 9 through its interaction with upstream Intron 8 sequences. **(A)** RIP assay was conducted to detect the binding ability of hnRNPA1 with LAS1L pre-mRNA, and the results showed that hnRNPA1 could directly bind to the pre-mRNA of LAS1L. **(B)** Graphical for pcDNA3.1-LAS1L-minigene. **(C, D)** AGE assays showed that the ratio of LAS1L-S/L in minigene was significantly reduced in hnRNPA1 knockdown group. **(E–H)** qPCR assays indicated that knockdown of hnRNPA1 with or without the minigene significantly increased the mRNA levels of LAS1L-L, thus reduced the ratio of LAS1L-S/L. **(I)** The RNA fragment sequence (wild-type and mutant) used in the RNA pulldown assay and its position in the LAS1L pre-mRNA, the red font represents the potential sites, and the blue font represents the mutated base. **(J, K)** Mutated minigene transfection assay showed that the ratio of LAS1L-S/L was significantly reduced after WT1-segment mutation or WT3-segment mutation compared with the minigene-control group, and the WT1-mutation site was the most significant. **(L–Q)** RNA pulldown assays showed that hnRNPA1 could bind with WT1 and WT3, but not WT2 or any other paired mutated sequences. Data are means ± SD (**P < 0.01, ***P < 0.001; ns P > 0.05).

The above observations suggest that hnRNPA1 can regulate the inclusion or exclusion of LAS1L exon 9. To further test whether hnRNPA1 regulates AS of LAS1L by directly binding pre-mRNA, we conducted RIP assays, and the results showed that hnRNPA1 could directly bind to the pre-mRNA of LAS1L (**Figure 5A**). To further illustrate whether hnRNPA1 regulates AS of LAS1L by directly binding to the region of exon 8 to exon 10 of LAS1L pre-mRNA, we constructed a minigene fragment corresponds to human LAS1L which contained the exons 8, 9, 10 and the flanking introns of exon 9 (350-nt upstream and 301-nt downstream) (**Figure 5B**). Semi-quantitative RT-PCR analysis and AGE assays showed that the ratio of LAS1L-S/L in minigene was significantly reduced in A549 and H1299 cells of knockdown hnRNPA1 compared with normal control cells (**Figures 5C–D**). Equally, the qPCR assays also indicated that knockdown of hnRNPA1 with or without the minigene significantly increased the mRNA levels of LAS1L-L, thus reduced the ratio of LAS1L-S/L (**Figures 5E–H**).

Previous studies have reported that hnRNPA1 has a more robust binding ability to UAGGGU, and a relatively moderate binding ability to UGGGGU and UAGAGU (23–25). To determine the specific binding site of hnRNPA1 on the pre-mRNA of LAS1L, we used bioinformatics tools (RBP map) to predict the potential hnRNPA1 binding sites on the sequence region of exon 8 to exon 10. As shown in **Figure 5I**, 3 integrated potential hnRNPA1 binding sites (WT1, WT2 and WT3) were eventually identified. Interestingly, these potential binding sites were all located in the intron 8 region rather than any other region. To determine the interactions between hnRNPA1 and those potential binding sites, we performed site-directed mutagenesis of the potential sequence based on the original pcDNA3.1-LAS1L-minigene plasmid vector (**Figure 5I**). The qPCR results indicated that the ratio of LAS1L-S/L was significantly reduced after WT1-segment mutation or WT3-segment mutation compared with the minigene-control group, and the WT1-mutation site was the most significant. However, there were no significant effects after WT2-segment mutation (**Figures 5J–K**). To further determine the direct binding ability between hnRNPA1 and the potential binding sites, RNA-pulldown assays were performed in A549 cells. The 3’biotin-labled RNA oligomers of the potential binding sequences (WT1, WT2 and WT3) and paired mutated binding sequences (M1, M2 and M3) were synthesized (**Figure 5I**). RNA pulldown assays showed that hnRNPA1 could bind with WT1 and WT3, but not WT2 or any other paired mutated sequences (**Figures 5L–Q**). It is noteworthy that hnRNPA1 has a more robust binding ability to WT1 sequences than WT3 sequences, which is consistent with the above results. Combined with the above results, we concluded that hnRNPA1 can directly bind to the WT1 and WT2 sites of intron 8 to regulate the AS of LAS1L exon 9.

## Discussion

As a fundamental mechanism for post-transcriptional gene regulation, AS is performed in more than 95% of human multiexon genes, greatly increasing the diversity and complexity of the human proteome (26, 27). However, AS regulation also increases the complexity of disease states in a sense, especially tumors (28). In this study, we clarified that RNA-binding protein hnRNPA1 as an alternative splicing modulator can inhibit NSCLC metastasis and EMT transition. More importantly, our data showed that hnRNPA1 depletion can inhibit exclusion of LAS1L exon 8 to produce the LAS1L-L protein isoform, thus promoting EMT transition and metastasis capacity of lung cancer cells *in vivo*. Thus, these results nominate hnRNPA1 as a splicing regulator for inhibition of EMT and tumor metastasis, highlighting the crucial role of hnRNPA1 in regulating lung cancer progression.

It has been reported that hnRNPA1 can regulate tumor metastasis and EMT transition through a variety of different molecular mechanisms. However, things are not quite that simple as we think. For instance, it is reported that hnRNPA1 can act as an intermediate molecule to further promote the metastasis and of hepatocellular carcinoma (11) and gastric cancer (10). Differently, a study clearly demonstrated that hnRNPA1 and KHSRP can synergistically participate in the AS regulation of epithelial factors and thus block the transition process of EMT (14, 15), while two other studies also showed that hnRNPA1 can acts as a translational repressor or directly inhibit the production of ΔRon to further restrain the EMT transition progress (16, 29). These studies have been carried out in a variety of different tumors and have drawn different conclusions, while the role of hnRNPA1 in lung cancer metastasis has not been reported, and the differences in these conclusions may be due to tumor heterogeneity. In this study, our results showed that hnRNPA1 can indirectly regulates tumor metastasis and EMT by regulates the AS of LAS1L pre-mRNA except for the direct AS regulation of the epithelial factors, which provided different understanding and more theoretical basis for the regulation of hnRNPA1 in tumor metastasis. Interestingly, the number of cases in which hnRNPA1 expression was up-regulated (13 cases) and down-regulated (12 cases) was basically equal in the lung cancer clinical samples collected, and no correlation was found between hnRNA1 expression and TNM staging. On the one hand, considering the limitations of the sample size, we expect to increase the sample size in subsequent studies to clarify the correlation between hnRNPA1 expression and clinical parameters. On the other hand, it has been reported that the nucleocytoplasmic relocalization of hnRNPA1 may occur under stress (30) or epigenetic modification (31), which will further affect the downstream splicing regulation of hnRNPA1. Nucleocytoplasmic shuttling is one of the crucial mechanisms of hnRNPA1, which is a very meaningful research direction, and the study on this direction may be the key to clarify the correlation between hnRNPA1 and clinical parameters.

HnRNPA1 protein can be functionally divided into three domains: RNA binding domain, RGG box domain composed of repeated sequences of Arg-Gly-Gly tripeptide, and nucleoplasmic shuttle domain (32). Dr. Branche’s studies firstly showed that hnRNPA1 has a most robust binding ability to UAGGGU instead of UAGGGA (24). Subsequently, many other studies have shown that hnRNPA1 can also bind to sequences of UAGG, GGGU, UAGAGA or UGGGGU (25, 33, 34). Moreover, several studies have confirmed that hnRNPA1 specificity concentrated on the 5 ‘-YAG-3’ motif, but its binding affinity with the RNA target containing the motif, which mainly depend on the context characteristics that tune hnRNPA1-RNA molecular recognition, varies greatly (34–37). However, the characteristic binding sequence of HRNNPA1 and its complex molecular mechanism have not been clearly concluded. Therefore, identifying the specific sequence in which hnRNPA1 directly binds to will provide a crucial theoretical basis to exploring the molecular mechanism by which hnRNPA1 regulates AS. In this study, the binding sequence of hnRNPA1 to LAS1L pre-mRNA was clearly verified by RNA-pulldown assay. Results showed that the WT1 sequence (AAGG ***UAGGGU*** UCUGGGGGCUCACUGCACCCU ***UAGGGU*** UCUG) which contain two segments of UAGGGU was proved to have a more robust binding ability with hnRNPA1, while the WT3 sequence (CAGC ***UGGGGU*** GGGCUGGGGGGCCCUGGGU ***UGGGGU*** GUGA) which contain two segments of UGGGGU also has a moderate binding ability with hnRNPA1. Interestingly, the WT2 sequence (GGAA ***UGGGGUGGGGU*** GGGA ***UGGGGUGGGGU*** CGGG) which contain two consecutive segments of UGGGGU has a very week binding ability with hnRNPA1.The M2 sequence derived from site-directed mutation of WT2 has the same weak binding ability compared with the WT2, which meant that the UGGGGUGGGGU fragment held together by two consecutive segments of UGGGGU lost its robust binding ability to hnRNPA1 instead compared with the single fragment of UGGGGU. One explanation may be that there are many continuous guanosines outside the targets of WT1 (AAGG ***UAGGGU*** UCU ***GGGGG*** CUCACUGCACCCU ***UAGGGU*** UCUG) and WT3 (CAGC ***UGGGGU*** GGGCU ***GGGGGG*** CCCUGGGU ***UGGGGU*** GUGA) sequences, while WT2 sequences do not have this structure.

LAS1L is an indispensable member of ribosomal biogenesis. More and more studies have shown that ribosomal biogenesis is significantly related to tumor progression (38–41), including tumor metastasis and EMT transition. For instance, Dr. Vincent’s studies show that the induction of ribosome biogenesis is a general feature of the EMT program and can promote the cellular plasticity and migration (42). Similarly, except for contributing to the ribosomal biogenesis, LAS1L has also been reported to be involved in tumor cell proliferation (18), metastasis (20) and autophagy (19). Among them, Dr. Samant ‘s research shows that knockdown of LAS1L leads to a sharp reduction in tumorigenic and metastatic properties in triple-negative breast cancer (20), which is similar to Dr. Vincent’s conclusion. In this study, we found that the regulation of LAS1L on tumor metastasis and EMT transition is dominated by LAS1L-L rather than LAS1L-S. One explanation is that LAS1L-L, which has a full-length peptide chain structure compared to LAS1L-S, may activate/inhibit different downstream molecules/pathways, and thereby produce completely different biological effects. However, our understanding and basis are still insufficient, and more research should be carried out to clarify its complex molecular mechanism.

In summary, our findings reveal the tumorigenic effects of hnRNPA1 and its downstream LAS1L- isoform in lung cancer cell metastasis and EMT transition. We also find that hnRNPA1 can directly regulate the exon skipping of LAS1L exon 9, which further promote the tumor metastasis and EMT transition. In-depth study of the AS regulation mechanism and biological effects of hnRNPA1 can provide an important theoretical basis and new layer for lung cancer research.

## Data Availability Statement

The original contributions presented in the study are included in the article/**Supplementary Material**. Further inquiries can be directed to the corresponding authors.

## Author Contributions

PH: Conceptualization, Methodology, Software, Writing- Original draft preparation. PC: Data curation, JY: Visualization, Investigation. KK and SH: Investigation, Supervision. LL: Supervision. YD: Methodology, Validation. FL and BZ: Conceptualization, Supervision. All authors contributed to the article and approved the submitted version.

## Conflict of Interest

The authors declare that the research was conducted in the absence of any commercial or financial relationships that could be construed as a potential conflict of interest.

## Publisher’s Note

All claims expressed in this article are solely those of the authors and do not necessarily represent those of their affiliated organizations, or those of the publisher, the editors and the reviewers. Any product that may be evaluated in this article, or claim that may be made by its manufacturer, is not guaranteed or endorsed by the publisher.

## Supplementary Material

Supplementary Figure 1
**(A)** Pearson correlation coefficient of the top 10 splicing proteins with LAS1L exon 9 expression in lung cancer from TCGA dataset. **(B)**The independent shRNA targeted against each splicing protein were verified by qPCR assays. **(C)**The potential effect of these splicing proteins on the regulation of LAS1L exon 9.

Supplementary Figure 2 Semi-quantitative RT-PCR analysis and AGE assays were conducted to detect the expression ratio of LAS1L-S and LAS1L-L in 29 pairs of tumor samples.
