# Supplementary figures and images for: Knockdown of hnRNPA1 Promotes NSCLC Metastasis and EMT by Regulating Alternative Splicing of LAS1L exon 9
Source: Front Oncol. 2022 Jun 23;12:837248. doi: 10.3389/fonc.2022.837248 (PMC9260696; doi:10.3389/fonc.2022.837248)

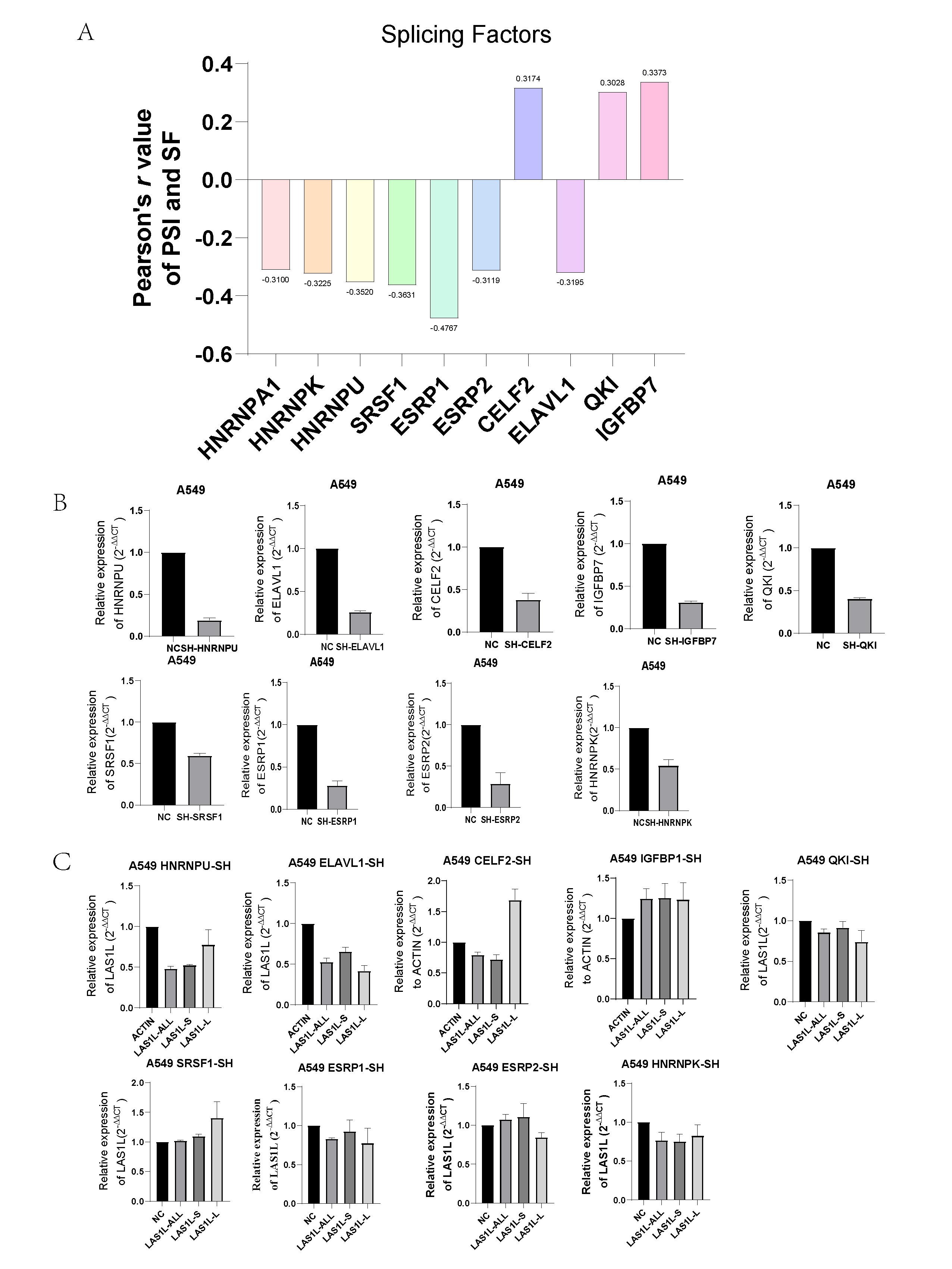

Supplement: Supplementary Figure 1 — (A) Pearson correlation coefficient of the top 10 splicing proteins with LAS1L exon 9 expression in lung cancer from TCGA dataset. (B)The independent shRNA targeted against each splicing protein were verified by qPCR assays. (C)The potential effect of these splicing proteins on the regulation of LAS1L exon 9. [file Image_1.tif]

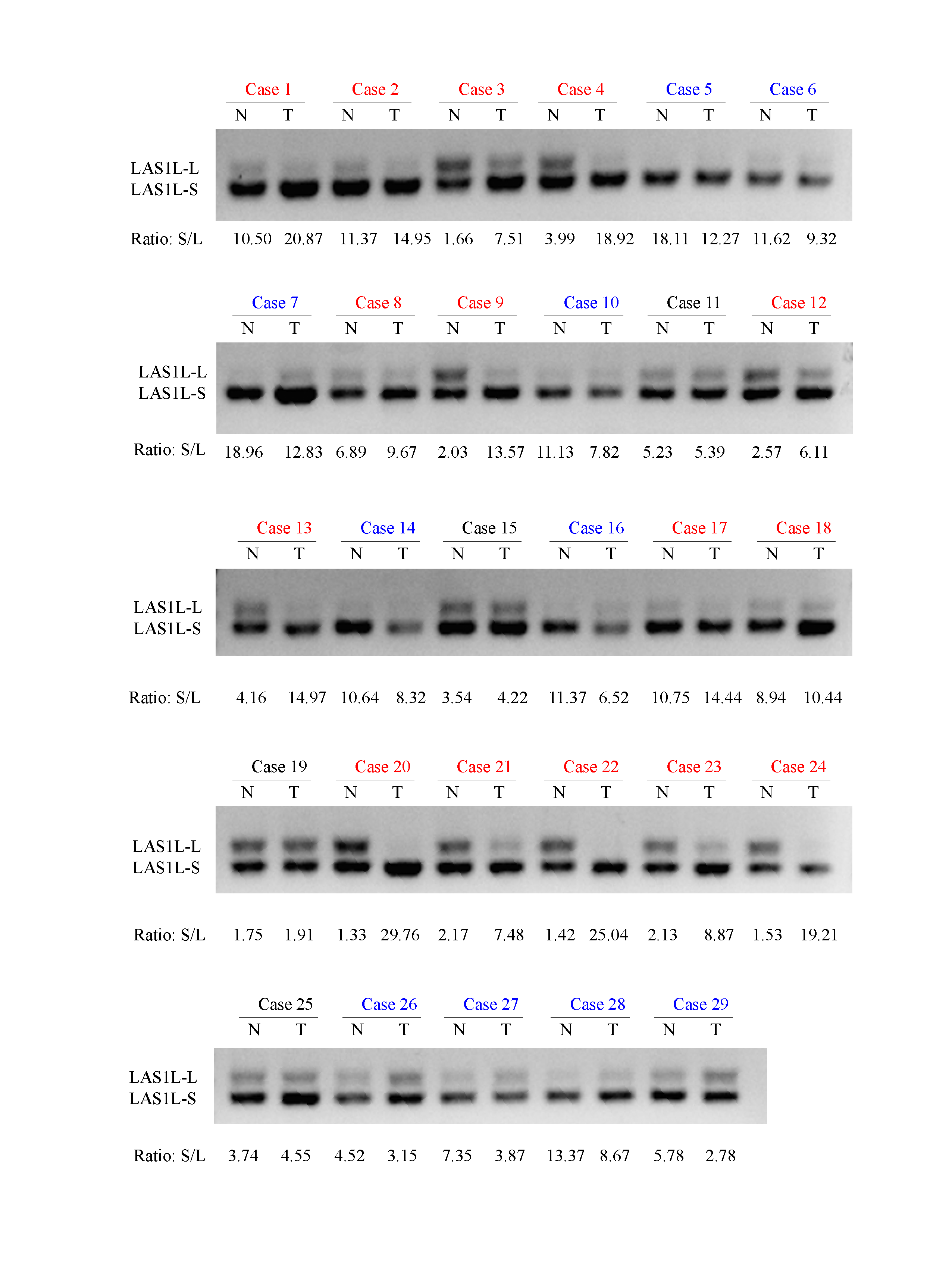

Supplement: Supplementary Figure 2 — Semi-quantitative RT-PCR analysis and AGE assays were conducted to detect the expression ratio of LAS1L-S and LAS1L-L in 29 pairs of tumor samples. [file Image_2.tif]
